# Burnout and Professional Engagement during the COVID-19 Pandemic among Nursing Students without Clinical Experience: A Cross-Sectional Study

**DOI:** 10.3390/jcm12155144

**Published:** 2023-08-06

**Authors:** Gustavo R. Cañadas, María José Membrive-Jiménez, María Begoña Martos-Cabrera, Luis Albendín-García, Almudena Velando-Soriano, Guillermo A. Cañadas-De la Fuente, Emilia Inmaculada De la Fuente-Solana

**Affiliations:** 1 Department of Didactic of Mathematics, Faculty of Education Science, University of Granada, Campus Universitario de Cartuja s/n, 18011 Granada, Spain; grcanadas@ugr.es; 2 Faculty of Health Sciences, University of Granada, Av. Ilustración 60, 18016 Granada, Spain; mjmembrive@ugr.es (M.J.M.-J.); gacf@ugr.es (G.A.C.-D.l.F.); 3 San Cecilio Clinical University Hospital, Andalusian Health Service, Av. del Conocimiento s/n, 18016 Granada, Spain; mariab.martos.sspa@juntadeandalucia.es (M.B.M.-C.); almudena.velando.sspa@juntadeandalucia.es (A.V.-S.); 4 Casería de Montijo Health Center, Granada-Metropolitan Health District, Andalusian Health Service, Calle Virgen de la Consolación 12, 18015 Granada, Spain; 5 Instituto de Investigación Biosanitaria (ibs.GRANADA), 18012 Granada, Spain; 6 Brain, Mind and Behaviour Research Center (CIMCYC), University of Granada, Campus Universitario de Cartuja s/n, 18011 Granada, Spain

**Keywords:** academic burnout, anxiety, COVID-19, depression, nursing students, online training

## Abstract

Burnout affects many healthcare professionals, especially nurses, causing serious health problems and disrupting the work environment. Academic burnout may also be experienced, leading students to feel unable to cope with their education. As a result, they may lose interest and even consider abandoning their studies. Hence, burnout syndrome can affect both the mental health and the professional future of those affected. To evaluate academic burnout in nursing students who had no clinical experience before starting their practical training, a cross-sectional study involving 212 third-year nursing students at the University of Granada was conducted. Data were collected using the Granada Burnout Questionnaire, the Utrecht Work Engagement Scale, the NEO Five-Factor Inventory, the Hospital Anxiety and Depression Scale, and the Fear of CoronaVirus-19 Scale. High levels of burnout were present in 37.8% of the students. Moreover, 21.5% and 8.7% had borderline cases of anxiety or depression, respectively. Another 30.8% and 9.2%, respectively, were considered likely to present these conditions. According to the predictive models of burnout dimensions obtained, neuroticism is a predictor of all three burnout dimensions. Furthermore, anxiety, depression, extraversion, responsibility and engagement are predictors of some dimensions of the syndrome. Many nursing students present high levels of burnout, which is related to certain personality variables and to the presence of anxiety and/or depression. The level of professional engagement is inversely associated with the impact of burnout. The participants in this study have normalised their return to the pre-pandemic study routine (in-person classes), and fear of COVID-19 was not a significant predictor of any dimension of burnout.

## 1. Introduction

Burnout syndrome is considered an occupational disease and it is included in the International Statistical Classification of Diseases and Related Health Problems (ICD-11) published by the World Health Organisation (WHO) [1].

The conceptualisation of burnout by the WHO in 2019 [2] was a landmark in the international recognition of the syndrome since it was first described by Freudenberger in 1974 [3]. Burnout results from the imperfect management of chronic work stressors, composed of three dimensions: emotional exhaustion (EE) or a lack of energy and/or a depletion of emotional and physical resources; depersonalisation (D), i.e., mental detachment and the development of negative, cynical attitudes towards others; and scant feelings of personal accomplishment (PA), or the tendency to view oneself as incompetent and to have negative perceptions of the work performed [4,5].

Burnout can affect men and women at many levels. Moreover, it is a significant predictor of certain diseases and of physical and mental symptoms such as hypercholesterolaemia, type 2 diabetes, muscle pain, insomnia or depression. The syndrome can have a very negative impact, both on those directly affected and also on the persons to whom they are responsible and on the environment in which they work or study [6].

One of the population groups most commonly affected by burnout is that of healthcare personnel, especially nurses, since patient care and treatment imposes continual emotional stresses [7]. In this field, too, occupational factors such as care overload and long shifts, sociodemographic ones such as age and gender, and psychological ones such as the individual’s personality can all contribute to the development of the syndrome [8,9].

The prevalence of burnout syndrome among nurses has been studied in various countries and institutions. According to a meta-analysis carried out by Woo et al. [7], the worldwide prevalence of burnout symptoms among nurses is 11.23%, although researchers in specific countries have reported much higher figures; in Spain, for example, a burnout prevalence of 40% has been recorded [8].

Burnout can also appear earlier in life, for example, among university students. Academic burnout is defined as the circumstance in which a student feels incapable of facing the challenges that may arise, instead developing an attitude of negative criticism, disparagement and uninterestedness, together with doubts about their own ability to complete professional training [9]. The change from high school to university, the academic pressure imposed by obligations such as assignments and exams, financial problems or a lack of family support—any or all of these factors can trigger high levels of stress in students, sometimes leading to emotional exhaustion [10].

The practical training of nursing students is essential. In fact, studies confirm the importance of this training for their professional future, as it prepares them adequately to manage patients in different clinical contexts [11]. The relatively early appearance of the syndrome can also influence nursing students’ mental and physical health. Due to long academic days of university training and also complicated emotional situations, students may develop stress and emotional instability [12]. During the pandemic, there was an added problem, as students had more e-learning and less contact with patients [13]. Hence, job skills would be impaired and the likelihood of their abandoning the profession would increase [14].

Burnout is significantly present among students. With respect to the individual dimensions of the syndrome, the estimated prevalence of emotional exhaustion is 55.4%, that of depersonalisation, 31.6%, and that of reduced academic efficacy, 30.9% [15]. Within the different branches of healthcare, the prevalence of burnout among medical students ranges from 45% to 71%. Furthermore, the condition has been related to the appearance of psychiatric disorders and even suicidal ideas [16].

Nursing students are subject to considerable academic stress during their acquisition of professional knowledge and skills [17]. Various studies have been undertaken to determine the prevalence of burnout among these students. Among them, Galdino et al. [18] recorded a prevalence of 10.5% in Brazil, while studies in China have reported levels of over 30% [17,19]. In a review and meta-analysis conducted by Kong et al. [20], based on data from Spain, the United States, Italy, Brazil and Canada, the overall prevalence observed was 23%.

The main purpose of clinical practice is to introduce students to the practical environment of healthcare. This transition, prior to their becoming full-fledged professionals, is an essential aspect of medical training and the programme is often highly demanding [21]. However, the fact that they are not yet fully trained and experienced often provokes insecurity when students must deal with professionals and patients. The situation is stressful, and sometimes aggravated by external pressures or even intimidation [22].

The COVID-19 pandemic was a further source of negative stimuli. Indeed, the situation faced by nursing students, who faced the challenge of learning under conditions of a global pandemic, was unprecedented. The psychological impact of generalised uncertainty and widespread crisis, one that impacted severely on even the most experienced professionals, heightened feelings of anxiety, depression and burnout among nursing students [23]. Therefore, we believe the present analysis of a population of university students, most of whom will subsequently become nurses, is not only of theoretical interest but may provide the necessary understanding to help prevent the development of burnout in the early stages of nursing practice.

This paper studies certain aspects of academic burnout in a sample of nursing university students prior to beginning clinical practice during the recent COVID-19 pandemic. The goals are as follows: (a) to know the levels of academic burnout of these students; (b) to analyse the relationship between the syndrome and certain sociodemographic and psychological risk factor; and (c) to elaborate a risk profile regarding the above factors. In view of these considerations, the main aim of the study we describe is to evaluate the impact of academic burnout on a group of nursing students who had no clinical experience before beginning healthcare practices during the recent COVID-19 pandemic. We are not aware of previous research with the same objective, so we believe that the present paper provides useful information on nursing students’ first contact with patients. Our starting hypotheses are as follows: (1) a significant proportion of students have high levels of academic burnout and (2) aspects of personality and student engagement are predictive of these levels of academic burnout.

## 2. Materials and Methods

### 2.1. Participants

The only criterion for inclusion in the research was that the students were enrolled in the Nursing Degree at the University of Granada, just before the start of the clinical practice. All students who met this criterion were invited to participate. The study population consisted of 212 third-year nursing students at the University of Granada, selected by non-probabilistic sampling. A total of 84.9% were women and the mean age of all participants was 21.891 years (SD = 5.273).

### 2.2. Procedure

The study design was cross-sectional and predictive [24]. At the beginning of the academic year, during the COVID-19 pandemic, the researchers contacted students performing the in-person nursing practice associated with this degree course and invited them to participate in the study. The data obtained for this purpose were compiled on an ad hoc online platform during the first semester of 2021. All participants gave signed informed consent and were guaranteed confidentiality and anonymity regarding the data provided.

### 2.3. Instruments

All participants completed a questionnaire focused on socio-demographic data, their own health status and that of their immediate environment in relation to the COVID-19 pandemic. Some of the questions included in the questionnaire were as follows: have you had COVID-19?; has anyone close to you had COVID-19?; has anyone close to you died from COVID-19?; among others. The following measuring instruments, all validated, were then administered:

- The Granada Burnout Questionnaire for University Students (CBG-US) [25], composed of 26 items scored on a five-point Likert scale. The CBG-US measures three dimensions of burnout: EE, D and PA. Higher scores in EE and D, and low scores in PA suggest higher levels of burnout.
- The Utrecht Work Engagement Scale (UWES) [26]. This instrument examines 24 items, scored on a seven-point response scale, considering the following dimensions of professional engagement: absorption (full concentration and placid immersion in one’s own tasks), dedication (commitment to one’s own tasks, together with feelings of importance and enthusiasm) and vigour (energy and mental resilience). Higher scores in all subscales imply a higher level of engagement.
- Four of the five dimensions of the Spanish version of the NEO Five-Factor Inventory (NEO-FFI) [27], namely neuroticism, extraversion, agreeableness and conscientiousness. This instrument consists of 48 items scored using a five-point Likert response format. Each sub-scale (dimension) contains twelve items. Higher scores in the subscales indicate higher degree of showing these personality characteristics.
- The Hospital Anxiety and Depression Scale (HADS) as adapted for use with a Spanish population [28], with two subscales: anxiety (seven items) and depression (seven items). These fourteen items are scored on a four-point Likert scale [29]. Higher scores reflect a higher level of depression and anxiety.
- The Fear of CoronaVirus-19 Scale (FCV-19S), as adapted for use with a population of Spanish students [30]. This instrument consists of seven items, each scored on a five-point scale. Higher total score reflects higher fear towards COVID-19.

### 2.4. Ethical Considerations

The study was approved by the Ethics Committee of the University of Granada, and the ethical considerations of the Declaration of Helsinki [31] were complied with at all times. The data were processed in accordance with the provisions of Act 3/(5 December 2018), on Personal Data Protection and guarantee of digital rights.

### 2.5. Data Analysis

All statistical analyses were performed with SPSS.25 software, as follows. First, the descriptive statistics were calculated and a correlational analysis of the study variables was performed. Secondly, the proportion of students corresponding to each level of burnout severity was determined. Thirdly, the differences obtained were analysed in relation to the different dimensions of burnout and professional engagement, according to the COVID-19 variables considered. The t-Student statistic was used to perform the comparisons. In cases where the data did not meet the homogeneity of variance assumption, the Welch approximation was used. In cases that did not meet the normality assumption and where the sample of subjects in a particular comparison group was small, the Kruskal–Wallis non-parametric test was used. Finally, predictive models were obtained for the three dimensions of burnout, using the remaining study variables as predictors, with multiple linear regression, using the backwards steps procedure, for which the assumptions of normality, linearity, collinearity and heteroscedasticity were checked.

## 3. Results

### 3.1. Levels of Burnout and Description of Related Variables

A total of 30.7% of the study participants had had COVID-19, 55.7% had not, and 13.7% did not respond to this question. In 76.4% of cases, a relative had had the disease, versus 9.4% in which this was not the case (14.2% of the participants did not answer this question). An amount of 13.2% of the students reported that a friend or close relative had died of COVID-19. Long-term consequences of the disease, whether physical or psychological, were reported by 45.3% and 70.8% of participants, respectively.

The impact of anxiety and depression was analysed in accordance with the recommendations of the authors of the HADS [29]. In this respect, 21.5% and 8.7% of the students were classed as borderline cases of anxiety and depression, respectively, while 30.8% and 9.2%, respectively, were considered probable cases. Table 1 presents the descriptive statistics for all the study variables considered.

An overall classification of the participants according to the burnout severity experienced was obtained by combining the results obtained for each dimension of the syndrome, following the model proposed by Golembiewski et al. [32] (see Table 2). A total of 37.8% of the students were classed as phase 6, 7 or 8, corresponding to high levels of burnout.

### 3.2. Levels of Burnout and Engagement According to Variables Related to COVID-19

Various hypotheses were tested to identify differences in burnout and professional engagement, according to the COVID-19-related variables considered (see Table 3). Significant differences were obtained in EE between those who had or had not been infected with COVID-19 (those who had not presented higher levels of EE). Participants who were close to someone who had been infected obtained lower levels of D, higher EE and lower PA. Students who had suffered physical or psychological consequences from the disease presented higher EE and FCV. Those who reported physical consequences had significantly lower levels of PA.

A correlation analysis performed between the study variables revealed moderate and significant correlations in almost all cases (see Table 1). Thus, EE was positively and significantly related to NE, FCV, AN and DP, and negatively related to PA, EX, VI, DE and AB. D was significantly and positively related to AN and DP, and negatively related to PA, AG, EX, CO, VI, DE and AB. Finally, PA was significantly related to AG, EX, CO, VI, DE and AB, and negatively related to NE, AN and DP. Furthermore, FCV was significantly related to the levels of EE and NE.

### 3.3. Risk Factors and Predictive Models of Burnout Levels

Predictive models were obtained for each of the dimensions of burnout, in which the remaining variables considered were taken as predictors. All of these models were statistically significant. Thus, *F* (4, 157) = 17.804, *p* < 0.001, *R*^2^*_Adj._* = 0.295 in EE; *F* (4, 160) = 17.695, *p* < 0.001, *R*^2^*_Adj._* = 0.289 in D; and *F* (2, 161) = 81.811, *p* < 0.001, *R*^2^*_Adj._* = 0.498 in PA (see Table 4). NE was found to be a predictor of each of the dimensions of burnout. Moreover, AN, DP, EX, CO, VI and DE were each a predictor of some of the dimensions of the syndrome. 

## 4. Discussion

The aim of this study is to evaluate aspects of academic burnout in nursing students without clinical experience, i.e., they only have the theoretical and practical knowledge acquired in the classroom. As these students had not previously interacted with patients and were beginning their healthcare practice during the COVID-19 pandemic, we believe it useful to analyse the situation arising with respect to burnout during this phase of their training. In this paper, we study the burnout levels of these students, the relationship between academic burnout and other variables, and possible predictors of burnout dimensions.

The results obtained support the initial hypothesis; a high percentage of the participants present high levels of burnout. In some cases, the stress experienced and the traumatic events witnessed in the pandemic provoked post-traumatic stress disorder and burnout [33]. Indeed, the simple fact of treating patients infected by COVID-19 may favour the appearance of burnout and it is not uncommon that the expectation of facing COVID-19 in the near future is a source of stress and burnout [34]. Thus, our survey responses reflected very high levels of burnout (37.8%), which may be considered in line with expectations, as some pre-pandemic studies reported burnout levels exceeding 40% [35], with subsequent values rising to 66% [36].

In addition to stress, other factors such as overwork and the fear of contagion can push even experienced nurses beyond their ability to cope with the prolonged physical and mental strain [37]. It is not surprising, therefore, that the students in our study, especially those who had not been infected, suffered the same consequences. The fact that they had not been infected generated insecurity and fear, making EE strongly present. Analysis of this situation could help researchers and managers develop appropriate strategies to promote resilience in the future [38].

Undoubtedly, psychological and physical disorders such as depression, anxiety, endocrine disorders and insomnia were commonly experienced during the COVID-19 pandemic [39]. Indeed, rates of stress, anxiety and depression were higher among the students in our study than among experienced healthcare staff, due to their fear of becoming infected [40]. This fear was especially prevalent among those previously inclined towards negative feelings or neuroticism (NE), and who dealt with this condition through social distancing. The pandemic-related circumstances provoked a further consequence, that of burnout [41].

The results obtained in the research indicate that EE is significantly related to some aspects of personality (NE and EX), engagement dimensions, and levels of anxiety and depression. The burnout dimension of EE was very evident among the students, possibly due to their need to adapt to new types of learning, whilst maintaining social distancing, and therefore receiving a lower level of ‘hands-on’ experience [42]. This proximity deficit led some students to score highly for NE, their negative feelings made manifest as fear and anxiety. Accordingly, the level of professional engagement fell considerably [43], especially in terms of personal and work relationships, creating an adverse work environment for future practical experience [44]. Another important consideration is that students with problems of reduced self-esteem are more likely to be dissatisfied with their academic progress and may develop depressive symptoms [45]. For all of these reasons, many of the students consulted felt they were insufficiently equipped to manage patient care and were emotionally affected by the many pandemic-related deaths witnessed [46].

Students’ levels of D are significantly related to some personality characteristics (NE, AG and EX), some engagement dimensions (VI and AB) and participants’ levels of anxiety and depression. The burnout dimension of D arises from a perceived lack of resources with which to overcome adverse situations. Thus, the scant learning resources available and the pressure imposed by the pandemic heightened the prevalence of anxiety and depression among students [47], generating attitudes of generalised dissatisfaction [48] and reduced engagement [43]. The students who were less conscientious were especially likely to suffer from depression and were less empathetic with patients [49]. By contrast, those who were more friendly and extroverted were better equipped to have a good relationship with patients [50,51]. Nevertheless, this proximity was severely limited by the fear of contagion [40] and the limits imposed by social distancing [42].

Students’ PA levels are significantly related to some personality characteristics (NE, AG and EX), dimensions of engagement and participants’ levels of anxiety and depression. Among the students consulted, feelings of PA were also impacted by the pandemic, and those who had negative feelings in this respect believed themselves less academically effective. In addition, some students presented alexithymia (difficulty experiencing, identifying and expressing emotions) [19], which together with the required distancing from classmates and teachers [52], the fear of contagion [40] and exposure to adverse events further aggravated their anxiety and depression [50]. However, in some cases, PA was increased via the boost to professional engagement achieved, for example, by an interactive online-learning methodology [53] in which social relationships and creative work were encouraged [18]. Furthermore, students who are outgoing, responsible and friendly tend to be more productive [54] and are happy to ask others for help when necessary, which makes them less vulnerable to stressful situations such as those arising during the COVID-19 pandemic [55].

The second hypothesis we put forward at the beginning of the study was that some aspects of personality and student engagement predict the dimensions of academic burnout. In line with predictive models of burnout dimensions, the psychological variables considered were relevant to the students participating in our study, as has been found for experienced nurses. In addition, some variables are specifically relevant to the academic field [56,57,58]. Thus, NE is a predictor of all three dimensions of burnout. A high level of NE is correlated with a negative or uncontrolled emotional focus, poor coping skills and problems caused by impulsivity, thus impacting psychological resilience [59]. All of these consequences may provoke anxiety and depression [60], reduce professional engagement [41], impair academic performance and make the individual less well equipped to enter the world of work [61]. 

Among the conclusions drawn from our findings with respect to clinical practice, it seems that when online working methods are required, good relationships among students and between them and their teachers should be fostered. In this respect, social distancing has been shown to be detrimental in professions that require a practical approach to learning [62].

Finally, we must take into account the level of burnout and engagement with respect to variables related to COVID-19. There was a large EE in those students who went to their internships and did not become infected. This situation was a consequence of daily anxiety and stress because they were constantly thinking that they would be the next to become infected [63,64].

At the same time, there is the case of students who became infected. Several authors claim that COVID-19 infection can lead to an inflammatory process leading to psychological sequelae such as anxiety and depression [65]. Some authors also claim that the central nervous system dysfunction caused by COVID-19 increases the likelihood of emotional disorders and psychological sequelae [66]. If we add to this the fact that COVID-19 survivors experience the stigma of the disease and the fear of imminent death, the long-term impact of these disorders is very great [67]. Therefore, students who are infected by COVID-19, especially those with physical sequelae, suffer from psychological sequelae such as post-traumatic stress [68]. And, it is well known that stress, according to several studies, is a predictor of academic burnout [69,70,71].

This study has some limitations that should be taken into account when interpreting the results. The first thing to note is that the design of a cross-sectional study does not allow conclusions to be drawn about causal relationships between the variables analysed. For this reason, it is interesting to consider conducting a prospective longitudinal study in order to analyse the progression of burnout in nursing students. Secondly, the choice of a non-random sample is another limitation to be taken into account. However, as we wanted to analyse nursing students without clinical experience, the sample was small and difficult to randomise. For this reason, the results of this study should be taken with caution.

### Implications for Clinical Practice

Future research should focus on interventions that can prevent the development of burnout in nursing students. For this reason, it would be interesting to analyse which personality factors make student nurses more vulnerable to burnout. Finally, diagnostic tools should be developed based on objective parameters related to stress, as it is the main cause of burnout.

## 5. Conclusions

Among the student nurses consulted, 37.8% presented high levels of burnout, affecting personality variables and provoking anxiety and depression. Personality variables such as neuroticism, extraversion and responsibility, together with anxiety and depression, are predictors of the syndrome.

Preventive measures during the pandemic conditioned the way of teaching. Social distancing and lack of clinical experience contributed to burnout in nursing students.

Moreover, the three dimensions of professional engagement are significantly related to burnout and may be considered protective factors. Accordingly, attention should be paid to these factors, in conjunction with the above-mentioned psychological variables, in order to reduce the impact of the syndrome and/or prevent its appearance.

Finally, the study findings suggest that, with the return to in-person classes and activities, nursing students became accustomed to the situation, and fear of COVID-19 was not a significant predictor for any of the dimensions of burnout.

## Figures and Tables

**Table 1:** Descriptive statistics and correlations for the study variables (*n* = 212).

Variable	1	2	3	4	5	6	7	8	9	10	11	12	13
M	27.7	11.3	36.31	35.4	43.29	43.29	45.78	16.45	8.12	14.15	10.11	4.36	8.33
SD	5.36	3.54	5.35	9.57	5.55	7.64	6.41	4.66	4.18	3.54	3.81	3.67	4.07
1. EE	1												
2. D	0.11	1											
3. PA	−0.49 **	−0.28 **	1										
4. NE	0.46 **	0.23 **	−0.44 **	1									
5. AG	−0.07	−0.33 **	0.3 **	−0.27 **	1								
6. EX	−0.26 **	−0.46 **	0.45 **	−0.5 **	0.39 **	1							
7. CO	−0.01	−0.38 **	0.28 **	−0.35 **	0.33 **	0.29 **	1						
8. FCV	0.25 **	−0.05	−0.12	0.25 **	0.04	−0.07	0.14	1					
9. VI	−0.34 **	−0.18 *	0.51 **	−0.41 **	0.32 **	0.33 **	0.35 **	−0.04	1				
10. DE	−0.22 **	−0.28 **	0.66 **	−0.29 **	0.35 **	0.38 **	0.37 **	0.047	0.6 **	1			
11. AB	−0.19 *	−0.18 *	0.49 **	−0.2 **	0.34 **	0.18 *	0.35 **	0.06	0.7 **	0.66 **	1		
12. AN	0.35 **	0.4 **	−0.47 **	0.6 **	−0.28 **	−0.54 **	−0.35 **	0.15 *	−0.37 **	−0.38 **	−0.25 **	1	
13. DP	0.47 **	0.2 **	−0.33 **	0.75 **	−0.21 **	−0.35 **	−0.24 **	0.26	−0.32 **	−0.23 **	−0.14	0.61 **	1

EE = Emotional exhaustion; D = Depersonalisation; PA = Personal Accomplishment (low level); NE = Neuroticism; AG = Agreeableness; EX = Extraversion; CO = Conscientiousness; FCV = Fear of Coronavirus; VI = Vigour; DE = Dedication; AB = Absorption; DP = Depression; AN = Anxiety. ** The correlation is assumed to be significant at 0.01. * The correlation is assumed to be significant at 0.05.

**Table 2:** Classification of the participants according to the phase model of Golembiewski et al. (1986).

Phase	1	2	3	4	5	6	7	8
D	L	H	L	H	L	H	L	H
PA	H	H	L	L	H	H	L	L
EE	L	L	L	L	H	H	H	H
% (*n*)	7.4 (13)	8.0 (14)	21.7 (38)	12.6 (22)	12.6 (22)	16.6 (29)	16.6 (29)	4.6 (8)

EE = Emotional exhaustion; D = Depersonalisation; PA = Personal Accomplishment; L = Low; H = High.

**Table 3:** Results of the hypothesis tests of differences in burnout and engagement according to COVID-19-related variables.

Variable	Outcome	M (SD)	M (SD)	t (df)	*p*-Value
		No	Yes		
Infected by COVID-19					
	EE	28.28	26.7	1.98 (150.986)	0.049
	D	11.35	11.22	0.245 (177)	0.806
	PA	36.22	36.47	−0.319 (158.041)	0.75
	VI	8.23	7.92	0.466 (177)	0.642
	DE	14.24	14	0.439 (179)	0.661
	AB	10.47	9.44	1.758 (178)	0.08
	FCV	16.65	16.06	0.816 (180)	0.416
Friend or relative infected by COVID-19					
	EE	27.42	27.74	−0.243 (174)	0.808
	D	13.85	10.98	3.521 (177)	0.001
	PA	33.1	36.72	−2.909 (176)	0.004
	VI	7.55	8.19	−0.861 (30.328)	0.396
	DE	12.45	14.41	−2.389 (178)	0.018
	AB	9.6	10.2	−0.666 (177)	0.506
	FCV	17.15	16.38	0.547 (21.598)	0.59
Friend or relative died from COVID-19					
	EE	27.63	28.11	−0.427 (174)	0.67
	D	11.32	11.22	0.126 (177)	0.9
	PA	36.3	36.33	−0.026 (176)	0.98
	VI	8.16	7.89	0.308 (177)	0.759
	DE	14.06	14.7	−0.873 (179)	0.384
	AB	10.12	10	0.152 (178)	0.879
	FCV	16.46	16.36	0.108 (180)	0.914
Psychological effects					
	EE	23.72	39.55	−4.91 (173)	0.000
	D	10.97	11.36	−0.568 (176)	0.571
	PA	37.16	36.1	1.014 (175)	0.312
	VI	8.35	8.05	0.362 (176)	0.718
	DE	14.34	14.1	0.35 (178)	0.727
	AB	9.88	10.12	−0.333 (177)	0.74
	FCV	13.55	17.03	−3.934 (179)	0.000
Physical effects					
	EE	26.41	28.94	−3.201 (173)	0.002
	D	10.95	11.66	−1.338 (176)	0.183
	PA	37.64	35.17	3.151 (175)	0.002
	VI	8.38	7.92	0.72 (176)	0.473
	DE	14.36	14.03	0.624 (178)	0.533
	AB	9.76	10.41	−1.138 (177)	0.257
	FCV	15.67	17.13	−2.112 (179)	0.036

EE = Emotional exhaustion; D = Depersonalisation; PA = Personal Accomplishment; AB = Absorption; DE = Dedication; VI = Vigour; FCV = Fear of Coronavirus.

**Table 4:** Multiple regression models for each dimension of burnout syndrome.

Variable	*B*	*SE*	95% CI for *B*		*p*	β
			*LL*	*UL*		
Emotional Exhaustion						
Intercept	11.797	3.53				
Anxiety	0.273	0.131	0.014	0.533	0.039	0.21
Vigour	−0.306	0.094	−0.491	−0.121	0.001	−0.241
Conscientiousness	0.243	0.061	0.123	0.362	0.000	0.287
Neuroticism	0.146	0.058	0.031	0.26	0.013	0.269
Depersonalisation						
Intercept	25.835	2.933				
Extraversion	−0.152	0.037	−0.225	−0.078	0.000	−0.333
Conscientiousness	−0.142	0.04	−0.22	−0.063	0.000	−0.253
Depression	0.252	0.086	0.083	0.421	0.004	0.261
Neuroticism	−0.074	0.031	−0.136	−0.012	0.019	−0.202
Personal Accomplishment						
Intercept	28.68	1.913				
Dedication	0.895	0.086	0.725	1.066	0.000	0.598
Neuroticism	−0.141	0.032	−0.205	−0.078	0.000	−0.253

CI = Confidence Interval; *LL* = Lower Limit; *UL* = Upper Limit.

## Data Availability

The data presented in this study are available upon request to the corresponding author. The data are not publicly available due to the Organic Law 3/2018 on Personal Data Protection and guarantee of digital rights.
